# 5-Fluorouracil combined with CalliSphere drug-eluting beads or conventional transarterial chemoembolization for unresectable hepatocellular carcinoma: a propensity score weighting analysis

**DOI:** 10.1038/s41598-024-77531-2

**Published:** 2024-10-26

**Authors:** Min Wei, Pengwei Zhang, Chaofeng Yang, Menglin Luo, Chengxi Zeng, Yujie Zhang, Yang Li

**Affiliations:** 1 Sichuan Key Laboratory of Medical Imaging, Department of Radiology, The Affiliated Hospital of North Sichuan Medical College, Nanchong, 637000 China; 2 Sichuan Key Laboratory of Medical Imaging, Department of Ultrasound, The Affiliated Hospital of North Sichuan Medical College, Nanchong, 637000 China; 3 Sichuan Key Laboratory of Medical Imaging, Department of Nuclear Medicine, The Affiliated Hospital of North Sichuan Medical College, Nanchong, 637000 China

**Keywords:** Hepatocellular carcinoma, TACE, Drug-eluting beads, Propensity score analysis, Efficacy and safety, Cancer therapy, Tumour angiogenesis, Chemotherapy

## Abstract

This study aimed to assess the effectiveness and safety of 5-Fluorouracil (5-Fu) combined with conventional transarterial chemoembolization (cTACE) compared to 5-Fu combined with drug-eluting bead transarterial chemoembolization (DEB-TACE) using CalliSpheres for the treatment of unresectable hepatocellular carcinoma (HCC) using propensity score weighting methods. This retrospective analysis included 131 patients with HCC treated with 5-Fu combined with cTACE (5-Fu-cTACE group, n = 65) or DEB-TACE (5-Fu-DEB-TACE group, n = 66) at the Affiliated Hospital of North Sichuan Medical College from January 2019 to December 2022. Based on the baseline data and laboratory indicators, propensity score weighting was used to reduce confounding bias. Modified response evaluation criteria in solid tumors (mRECIST) were used to evaluate clinical efficacy. The primary endpoint was progression-free survival (PFS), and the secondary endpoints were the disease control rate (DCR), objective response rate (ORR) and adverse events (AEs). PFS was assessed using Kaplan‒Meier analysis and Cox proportional hazards models. The ORRs at 1 month (M1) after treatment in the 5-Fu-DEB-TACE group and 5-Fu-cTACE group were 90.9% and 76.9%, respectively (P = 0.029), while at this time, the DCRs were 93.9% in the 5-Fu-DEB-TACE group and 90.8% in the 5-Fu-cTACE group (P = 0.494). At 3 months (M3) after treatment, the 5-Fu-DEB-TACE group had a higher ORR (84.8% vs. 56.9%, P < 0.001) and DCR (84.8% vs. 72.3%, P = 0.08). The ORR at 6 months (M6) was also higher in the 5-Fu-DEB-TACE group than in the 5-Fu-cTACE group (72.7% vs. 50.8%, P = 0.01). The median PFS after treatment with 5-Fu-DEB-TACE was longer than that after treatment with 5-Fu-cTACE (11 months vs. 6 months) (P = 0.004). Cox proportional hazards regression analysis indicated that 5-Fu-DEB-TACE (HR = 0.590, P = 0.044), Model for End-Stage Liver Disease (MELD) intermediate risk (HR = 2.470, P = 0.010), BCLC stage B (HR = 2.303, P = 0.036), BCLC stage C (HR = 3.354, P = 0.002) and ascitic fluid (HR = 2.004, P = 0.046) were independent predictors of PFS. No treatment-related deaths occurred in this study. The 5-Fu-DEB-TACE group had a greater incidence of abdominal pain (72.7% vs. 47.7%, P = 0.003). However, the incidence of postoperative elevated transaminase levels was higher in the 5-Fu-cTACE group (83.1% vs. 66.6%, P = 0.031). Subgroups analysis showed patients receiving 5-Fu-DEB-TACE have better PFS compared to those receiving 5-Fu-cTACE in the BCLC stage A group (P = 0.0093), BCLC stage B group (P = 0.0096), multifocal group (P = 0.0056), Child-Pugh stage A group (P<0.001), non- extrahepatic metastasis group (P = 0.022), non-vascular invasion group (P = 0.0093), and the group with a largest tumor diameter ≥ 5 cm (P = 0.0048). At M1, M3, and M6, patients with preserved liver function and in some cases of low tumor burden had higher Objective Response Rate (ORR) and Disease Control Rate (DCR) (P < 0.05). Compared with 5-Fu-cTACE, 5-Fu-DEB-TACE has superior therapeutic efficacy, prolongs PFS, and reduces hepatotoxicity. However, it is associated with an increased incidence of postoperative abdominal pain.

## Introduction

Hepatocellular carcinoma (HCC) is a common malignant tumor with the sixth highest incidence and the third highest mortality rate in the world^1^. At present, the best treatment for hepatocellular carcinoma is still surgical resection, but most patients are in the middle or advanced stage of the disease and miss the opportunity for surgery. For patients with unresectable HCC, especially those with intermediate-stage HCC and early-stage HCC for which other treatments are unsuccessful or not feasible, transcatheter arterial chemoembolization (TACE) is recognized as the recommended treatment in guidelines^2^. In contrast to the normal hepatic parenchyma, which is supplied by the portal vein (75%) and hepatic artery (25%), approximately 95% of hepatocellular carcinomas are mainly supplied by the hepatic artery. Therefore, the main principle of TACE is to block the artery providing the blood supply to the tumor while conducting local chemotherapy, which reduces the damage to the normal liver parenchyma compared with systemic chemotherapy. Two main types of TACE are currently available at present. One is conventional TACE (cTACE), which involves the use of an iodized oil-based emulsion (lipiodol oil) with chemotherapeutic drugs. cTACE therapy has several significant limitations^3–5^: (1) cTACE has a greater incidence of postembolization syndrome and has been associated with severe pain; (2) drug release in cTACE is relatively uncontrollable; (3) the embolic emulsion may be washed out; and (4) increased exposure to nontargeted drugs has been noted. Drug-eluting bead TACE (DEB-TACE), which uses new drug-eluting beads to load chemotherapeutic drugs through mechanisms such as ion exchange or absorption, has been developed to address these issues achieve targeted drug delivery. These beads are then infused into the target tumor via catheters or microcatheters, allowing for the sustained and prolonged release of drugs over time. This approach maintains a high concentration of drugs within the tumor while minimizing systemic exposure.

DEB-TACE has been widely available since 2006. However, the efficacy and safety of these two types of TACE are still controversial. Several randomized controlled trials (RCTs) and prospective studies have shown no difference in the tumor response or safety between cTACE and DEB-TACE^6–9^. Numerous meta-analyses have shown divergent results, with some suggesting no significant difference in efficacy between these two treatment modalities^10,11^, while others suggest that patients treated with DEB-TACE have longer overall survival (OS) and fewer adverse events (AEs)^12–15^. Recently, several retrospective studies have also confirmed the advantages of DEB-TACE over cTACE^16,17^. CalliSphere microspheres (CSM), the first drug-elating microspheres developed in China, are composed of polyvinyl-based macromolecular cross-linked polymers. These microspheres are capable of loading positively charged chemotherapeutic agents, such as doxorubicin and oxaliplatin, and offer a broad range of selectable particle sizes from 100 to 1200 μm. Several clinical studies and trials have documented its satisfactory efficacy and safety profile^18–20^. Furthermore, compared to other types of microspheres, it exhibits superior tumor response characteristics^21^. 5-Fluorouracil is a first-line chemotherapy drug for the treatment of hepatocellular carcinoma. It can interfere with the DNA synthesis of cancer cells, thereby inhibiting the growth and division of tumor cells, and it can also disrupt tumor angiogenesis to restrict tumor growth by blocking the blood supply to the tumor^22^. Studies have shown that the combination of 5-Fu and other chemotherapeutic drugs is beneficial for improving treatment efficacy and addressing drug resistance^23^. A meta-analysis studied the efficacy of TACE combined with different chemotherapy agents showed that the efficacy outcomes of TACE combined with 5-Fu ranked higher than other chemotherapy agents^24^. Furthermore, combining external beam radiotherapy (RT) with 5-Fu has been explored to increase the local failure-free survival (LFFS) and overall survival (OS) for locally advanced HCC^25^. However, comparative studies of 5-Fu combined with DEB-TACE with CSM and cTACE are limited, and the differences in efficacy and safety between the two methods in HCC patients remain unclear. Furthermore, a consensus has not been reached in clinical practice on which TACE technique should be used for patients with unresectable hepatocellular carcinoma, and a lack of agreement on the clinical factors associated with the treatment response has been noted. This study sought to utilize propensity score weighting methods to balance confounding variables and compare the PFS of patients with unresectable HCC following 5-Fu combined with cTACE or DEB-TACE with that following CSM treatment while evaluating the ORR and DCR at 1, 3, and 6 months posttreatment, as well as analysing AEs and factors impacting prognostic outcomes.

## Materials and methods

### Patients and data collection

This study was approved by the Medical Ethics Committee of Affiliated Hospital of North Sichuan Medical College. Informed consent was waived due to the retrospective nature of the study, with the waiver also approved by the Medical Ethics Committee of Affiliated Hospital of North Sichuan Medical College. All methods employed were conducted in accordance with relevant guidelines and regulations. This retrospective study included 131 patients with unresectable HCC who received DEB-TACE or cTACE combined with 5-Fu treatment as first-line treatment; all patients were from the Affiliated Hospital of North Sichuan Medical College.

The objective of this study was to compare the short-term efficacy and safety of 5-Fu-DEB-TACE treatment and 5-Fu-cTACE treatment in patients with HCC in China. The inclusion criteria for this study were as follows: (1) unresectable HCC confirmed by imaging or pathological diagnosis; (2) Child‒Pugh class A or B liver function; (3) the target lesion in the liver had not received other treatments, such as surgical resection, ablation or TACE; and (4) at least one measurable lesion.

The exclusion criteria were as follows: (1) patients diagnosed with intrahepatic cholangiocarcinoma or mixed cell carcinoma; (2) patients whose tumor response could not be evaluated according to modified response evaluation criteria in solid tumors (mRECIST), whose lesion was less than 1 cm, whose lesion was not suitable for repeated measurement, or whose lesion did not show tumor enhancement on contrast-enhanced CT or MRI; (3) patients with diffuse or extensive distant metastasis and whose expected survival time was less than 3 months; (4) patients who were lost to follow-up without follow-up data; and (5) patients who switched between DEB-TACE and cTACE within 6 months.

After eligible patients were screened, the data of the patients were collected from the electronic medical records or medical records department, including the patient’s medical history, clinical characteristics, laboratory tests, tumor markers, previous treatment, equipment and drugs used during TACE treatment. Treatment efficacy was evaluated, adverse events were recorded, and progression-free survival was followed.

Baseline information of the patients was collected, including: (1) age and sex; (2) medical history—diabetes, hepatitis B (HB), and liver cirrhosis; (3) clinical characteristics—tumor location (unilobar or bilobar), tumor distribution (single or multiple), largest nodule size, vascular invasion, lymphatic metastasis, extrahepatic metastasis, Eastern Oncology Group (ECOG) performance status, Child–Pugh stage, Barcelona Clinic Liver cancer (BCLC) stage; albumin–bilirubin (ALBI) score, and Model for End-Stage Liver Disease (MELD); (4) laboratory indicators of liver function and kidney function—albumin (ALB), total bilirubin (TBIL), alanine aminotransferase (ALT), aspartate alanine aminotransferase (AST), alkaline phosphatase (ALP), serum creatinine (SCR), and International Normalized Ratio (INR); and (5) tumor markers—alpha-fetoprotein (AFP) and Protein Induced by Vitamin K Absence or Antagonist-II (PIVKA-II).

All patients underwent routine blood tests, routine biochemical tests, coagulation function tests, alpha-fetoprotein detection and enhanced CT or enhanced MR imaging before treatment. Before treatment, all patients were informed of the purpose of surgical treatment, possible postoperative complications were described to the patients and their families, and the informed consent form was signed. All operations were performed by qualified professionals above the attending physician who were proficient in DEB-TACE and cTACE. Before chemoembolization, angiography of the common hepatic artery and superior mesenteric artery was performed to evaluate vascular anatomy, tumor vessels, tumor extent, and portal vein patency.

### 5-Fu-DEB-TACE procedures

CalliSphere microspheres (CSMs, 100–300 μm, Hondray Jialisheng Biomedical Technology Co., Ltd., China) were used as the carrier and embolic agent during DEB-TACE treatment. Each vial of drug-eluting beads was loaded with 70 mg of epirubicin. First, a bottle of CSMs was gently shaken to evenly distribute the CSMs in the bottle. The CSMs were then extracted with a 20 mL syringe and allowed to stand for 1 to 2 min at room temperature until the CSMs were completely precipitated, after which the supernatant was removed. Epirubicin (Epirubicin Hydrochloride for Injection, Shandong New Time Pharmaceutical Co. Ltd., China) (70 mg) was diluted into a syringe. The syringes with drug-loaded microspheres and epirubicin were connected and mixed using a three-way valve. The mixture was gently shaken every 5 min until the CSMs were loaded with chemoembolization reagent, and the drug loading time was approximately 30–45 min. At the end of drug loading, the microspheres were suspended in sterile water for injection or in nonionic contrast medium at a ratio of 1:1. Overall, the final injectable volume per vial was approximately 20 ml.

The patient was placed in the supine position, the puncture site area was routinely disinfected and covered with towels, and local infiltration anaesthesia was applied. Using the Seldinger method, the catheter sheath was placed through the femoral artery through percutaneous puncture, and the catheter was placed in the celiac artery or common hepatic artery for DSA (Azurion 7 M20, Koninklijke Philips N.V., Netherlands). Angiography images were collected, including arterial phase, parenchymal phase and venous phase images. The imaging findings were analysed to determine the location, size, number and feeding arteries of the tumors. If the blood vessels in some areas of the liver are sparse or the tumor is not completely stained, angiography of the superior mesenteric artery, renal artery, left gastric artery, inferior phrenic artery, lumbar artery, etc., should be performed to determine the ectopic origin of hepatic artery or extrahepatic artery collateral vessels. Superselective catheterization was used for the tumor feeding artery. A 2.4 F microcatheter (Renegade STC 18 Microcatheter, Boston Scientific Corporation, Marlborough, MA, USA) was inserted coaxially into the tumor-feeding arteries for arterial infusion using 5-Fu (Fluorouracil for Injection, Shanxi Powerdone Pharmaceutical Co., Ltd., China) (0.75 g/m2) for 15 min. Subsequently, the injection speed of the drug-loaded microspheres was 1 ml/min, and the microspheres were maintained in a good distribution and uniform suspension state during the injection process. When the flow rate of the drug-loaded microspheres and contrast medium suspension in the tumor-feeding artery did not decrease within 3 to 4 cardiac cycles, it was regarded as the end point of embolization, and the injection was stopped. Angiography was repeated 5 to 15 min after stopping the injection, and if tumor staining still existed, embolization continued until the end point of embolization (disappearance of tumor staining) was reached. After the injection of 2 ml (1 bottle) of drug-loaded microspheres, the lesions that still did not reach the embolization endpoint were embolized with drug-loaded microspheres, blank microspheres or iodized oil with a single embolization dose of no more than 4 ml until the embolization endpoint.

### 5-Fu-cTACE procedures

The procedure was identical to 5-Fu-DEB-TACE, with hepatic angiography performed in the same manner as described above, followed by superselection to the tumor-feeding artery. Subsequently, 30 mg of lobaplatin (Lobaplatin for Injection, Hainan Changan International Pharmaceutical Co., Ltd., China) and 40 mg of epirubicin were dissolved in a nonionic contrast agent. Then, they were mixed thoroughly with iodized oil to form an emulsion. The amount of iodized oil was determined according to the size and number of tumors and the richness of the arterial blood supply. When superselective embolization was performed at the segmental or subsegmental level, gelatine sponge embolization was performed after the injection of iodized oil emulsion until the embolic material was completely blocked to the tip of the catheter. For less selective lobar hepatic chemoembolization, the endpoint of embolization is the “dry branch” shape of the feeding artery; namely, the small tumor-feeding artery is embolized while preserving the patency of the hepatic segment or lobar artery to facilitate the second embolization^26^.

### Follow-up assessments

Postoperative enhanced CT or enhanced MRI and laboratory tests (alpha-fetoprotein, blood cell count, liver function, coagulation function, etc.) were reviewed monthly. When the tumor was still active or relapsed, TACE was repeated. All imaging data were reviewed by two independent radiologists with > 4 years of experience, and disagreements were resolved by consensus. The primary endpoint was progression-free survival (PFS), and the secondary endpoints were the disease control rate (DCR), objective response rate (ORR) and incidence of adverse events. The tumor response was evaluated according to mRECIST, and the tumor response was divided into a complete response (CR), partial response (PR), stable disease (SD) and progressive disease (PD). CR was defined as all lesions on enhanced arterial phase imaging disappear. PR was defined as the sum of the diameters of target lesions (enhanced arterial phase imaging) decreases by ≥ 30%. SD was defined as neither meeting the criteria for PR nor PD. PD was defined as the sum of the diameters of target lesions (enhanced arterial phase imaging) increases by ≥ 20%, or new lesions appear. The ORR was defined as the percentage of patients who achieved CR or PR among all patients, and the DCR was defined as the percentage of patients who achieved CR, PR or SD. PFS was defined as the time from the initiation of treatment to the occurrence of disease progression^27^. All AEs were recorded and assessed using the common terminology criteria for adverse events (CTCAE v5.0)^28^.

### Statistical analysis

Normally distributed continuous variables are described as the means ± standard deviations, nonnormally distributed continuous variables are described as medians/interquartile ranges, and categorical variables are described as numbers/percentages. Fisher’s exact probability test was used for categorical variables, and a t test was used for continuous variables. The independent variables included in the propensity score model were age, sex, cirrhosis status, alpha-fetoprotein status, PIVKAII score, ECOG score, Child‒Pugh grade, MELD score, ALBI score, number of tumors and tumor size. Baseline patient and tumor characteristics were compared after propensity score weighting using contingency tables and Fisher’s exact test (two parameters) or chi-square test (> two parameters), respectively, to confirm the comparability of the two groups. The Kaplan‒Meier method was used to evaluate the progression-free survival and overall survival of the two groups, and *P* < 0.05 was considered to indicate statistical significance. A propensity score-weighted Cox proportional hazards regression model was used to analyse the prognostic factors. AEs after TACE were compared using Fisher’s exact test. All the statistical analyses were performed using R 3.4.2 (2023, R Core Team, Vienna, Austria). Propensity score weighting was performed using the add-in R package “RISCA”, and the survival analysis was performed using the “survminer” package.

## Results

### Patients’ baseline characteristics

**Fig. 1:**
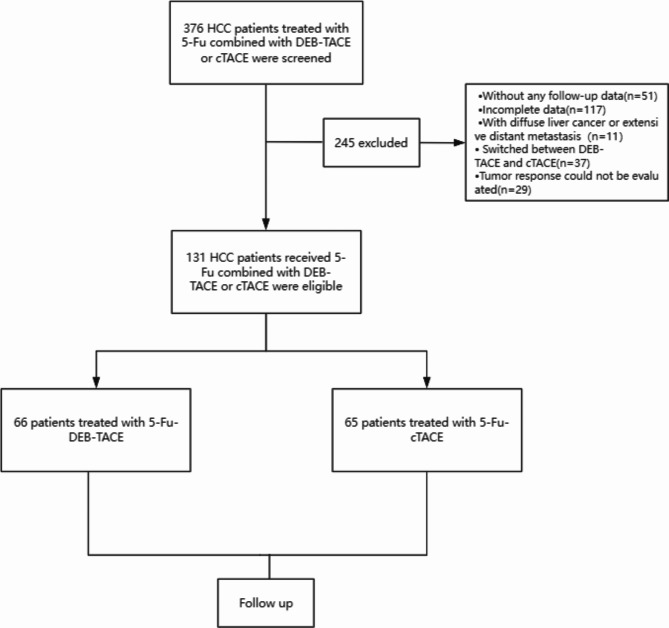
Flowchart of the trial profile. HCC, hepatocellular carcinoma; DEB-TACE, drug-eluting beads trans-arterial chemoembolization; cTACE, conventional trans-arterial chemoembolization; 5-Fu, 5-Fluorouracil.

A total of 376 patients with liver cancer treated with 5-Fu combined with DEB-TACE or cTACE were initially screened, and 245 patients were excluded (including 51 patients without any available follow-up data, 117 patients with incomplete data, 11 patients with diffuse liver cancer or extensive distant metastasis, and 37 patients who switched between DEB-TACE and cTACE within 6 months). The tumor response could not be evaluated in 29 patients. (Fig. 1). Ultimately, 131 patients with pure HCC treated with 5-Fu combined with DEB-TACE or cTACE as the first-line therapy were included in the analysis, including 66 patients treated with 5-Fu-DEB-TACE and 65 patients treated with 5-Fu-cTACE.

The baseline characteristics of the patients before propensity score weighting are shown in Table 1. Statistically significant differences were observed between the two groups in liver cirrhosis (*P* = 0.01), ECOG score (*P* = 0.001), BCLC stage (*P* = 0.008), and lymph node metastasis (*P* = 0.008). The baseline characteristics of the two groups after propensity score weighting are shown in Table 2, and no significant differences in the liver cirrhosis status, ECOG score, BCLC classification, lymph node metastasis status, sex, age or other aspects were observed (*P* > 0.05).

**Table 1:** Baseline patient characteristics before propensity score weighting.

Parameter	5-Fu-DEB-TACE(*n* = 66)	5-Fu-cTACE(*n* = 65)	*p*-Value
Gender			0.459
Male	57(86.4%)	52(80%)	
Female	9(13.6%)	13(20%)	
Age(years)	57.71 ± 11.99	58.77 ± 9.93	0.584
Diabetes Yes No	5(7.6%) 61(92.4%)	10(15.4%) 55(84.6%)	0.259
History of HB Yes No	46(59.7%) 20(30.3%)	53(81.5%) 12(18.5%)	0.169
Liver Cirrhosis Yes No	38(57.6%) 28(42.4%)	52(80%) 13(20%)	0.01
TBIL(µmol/L)	22.23 ± 13.09	21.87 ± 9.25	0.855
SCR(µmol/L)	66.97 ± 13.38	67.00 ± 13.03	0.988
ALB(g/L)	40.92 ± 4.84	40.64 ± 5.58	0.755
INR	1.11 ± 0.14	1.12 ± 0.14	0.762
AFP(µg/L) ≥ 400 <400	24(36.4%) 42(63.6%)	24(36.9%) 41(63.1%)	1.000
PIVKA-II(mAU/ml) ≥ 40 <40	54(81.8%) 12(18.2%)	54(83.1%) 11(16.9%)	1.000
Ascites Yes No	16(24.2%) 50(75.8%)	13(20%) 52(80%)	0.708
MELD Low risk Medium risk	65(98.5%) 1(1.5%)	64(98.5%) 1(1.5%)	1.000
ALBI Level 1 Level 2	39(59.1%) 27(40.9%)	38(58.5%) 27(41.5%)	1.000
Child-pugh Stage A B	57(86.4%) 9(13.6%)	59(90.8%) 6(9.2%)	0.605
ECOG performance status 0 1 2	53(80.3%) 9(13.6%) 4(6.1%)	33(50.8%) 26(40.0%) 6(9.2%)	0.001
BCLC Stage A B C	23(34.8%) 28(42.4%) 15(22.7%)	11(16.9%) 24(36.9%) 30(46.2%)	0.008
Largest nodule size (cm) ≥ 5 <5	55(83.3%) 11(16.7%)	48(73.8%) 17(26.2%)	0.266
Tumor multiplicity Unifocal Multifocal	33(50%) 33(50%)	28(43.1%) 37(56.9%)	0.536
Vascular Invasion Yes No	15(22.7%) 51(77.3%)	24(36.9%) 41(63.1%)	0.113
Lymphatic Metastasis Yes No	3(4.5%) 63(95.5%)	14(21.5%) 51(78.5%)	0.008
Extrahepatic metastasis Yes No	4(6.1%) 62(93.9%)	12(18.5%) 53(81.5%)	0.057

DEB-TACE, drug-eluting beads trans-arterial chemoembolization; cTACE, conventional trans-arterial chemoembolization; 5-Fu, 5-Fluorouracil; HB, hepatitis B; SCR, serum creatinine; ALB, albumin; INR, International Normalized Ratio; AFP, alpha-fetoprotein; PIVKA-II, Protein Induced by Vitamin K Absence or Antagonist-II; MELD, Model for End-Stage Liver Disease; ALBI, Albumin-Bilirubin; ECOG, Eastern Oncology Group; BCLC, Barcelona Clinic Liver cancer.

**Table 2:** Baseline patient characteristics after propensity score weighting.

Parameter	5-Fu-DEB-TACE(*n* = 66)	5-Fu-cTACE(*n* = 65)	*p*-Value
Gender			0.858
Male	103.0(83.5%))	96.0(82.1%	
Female	20.3(16.5%)	20.9(17.9%)	
Age(years)	58.77 ± 10.93	58.67 ± 9.76	0.959
Diabetes Yes No	12.9(10.5%) 110.4 (89.5%)	15.5(13.2%) 101.4(86.8%)	0.705
History of HB Yes No	91.8(74.4%) 31.6(25.6%)	94.2(80.6%) 22.7(19.4%)	0.473
Liver Cirrhosis Yes No	80.5(65.2%) 42.9(34.8%)	87.2(74.6%) 29.7(25.4%)	0.343
TBIL(µmol/L)	21.54 ± 12.60	22.18 ± 9.61	0.770
SCR(µmol/L)	65.00 ± 13.13	68.10 ± 13.90	0.269
ALB(g/L)	41.18 ± 4.59	40.64 ± 5.81	0.615
INR	1.13 ± 0.14	1.11 ± 0.14	0.674
AFP(µg/L) ≥ 400 <400	44.5(36.1%) 78.8(63.9%)	38.2(32.6%) 78.7(67.4%)	0.772
PIVKA-II(mAU/ml) ≥ 40 <40	96.0(77.8%) 27.4(22.2%)	96.2(82.3%) 20.7(17.7%)	0.605
Ascites Yes No	31.4(25.4%) 92.0(74.6%)	16(24.2%) 50(75.8%)	0.631
MELD Low risk Medium risk	121.4(98.4%) 1.9(1.6%)	115.0(98.3%) 1.9(1.7%)	0.970
ALBI Level 1 Level 2	74.6(60.5%) 48.7(39.5%)	69.7(59.6%) 47.2(40.4%)	0.928
Child-pugh Stage A B	108.3(87.8%) 15.0(12.2%)	102.5(87.7%) 14.4(12.3%)	0.987
ECOG performance status 0 1 2	83.7(67.9%) 32.0(25.9%) 7.6(6.2%)	72.2(61.7%) 35.1(30.0%) 9.7(8.3%)	0.804
BCLC Stage A B C	41.1(33.3%) 44.9(36.4%) 37.3(30.2%)	29.0(24.8%) 45.7(39.1%) 42.1(36.1%)	0.661
Largest nodule size (cm) ≥ 5 <5	99.1(80.3%) 24.3(19.7%)	89.0(76.1%) 27.9(23.9%)	0.638
Tumor multiplicity Unifocal Multifocal	62.2(50.4%) 61.1(49.6%)	56.3(48.1%) 60.7(51.9%)	0.825
Vascular Invasion Yes No	37.0(30.0%) 86.4(70.0%)	37.5(32.1%) 79.4(67.9%)	0.113
Lymphatic Metastasis Yes No	11.3(9.2%) 112.0(90.8%)	17.3(14.8%) 99.6(85.2%)	0.460
Extrahepatic metastasis Yes No	10.5(8.5%) 112.8(91.5%)	15.8(13.5%) 101.1(86.5%)	0.454

DEB-TACE, drug-eluting beads trans-arterial chemoembolization; cTACE, conventional trans-arterial chemoembolization; 5-Fu, 5-Fluorouracil; HB, hepatitis B; SCR, serum creatinine; ALB, albumin; INR, International Normalized Ratio; AFP, alpha-fetoprotein; PIVKA-II, Protein Induced by Vitamin K Absence or Antagonist-II; MELD, Model for End-Stage Liver Disease; ALBI, Albumin-Bilirubin; ECOG, Eastern Oncology Group; BCLC, Barcelona Clinic Liver cancer.

### Tumor response

**Fig. 2:**
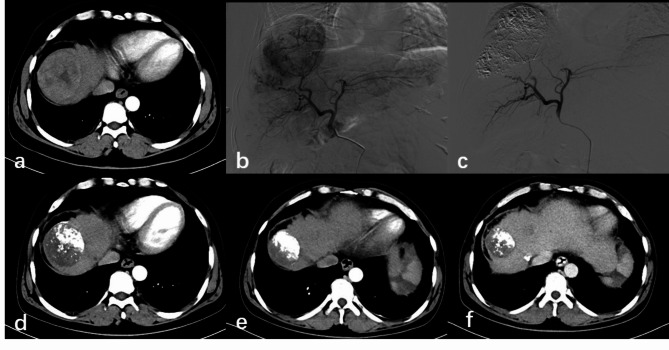
A 50-year-old male patient with hepatocellular carcinoma received DEB-TACE combined with CSMs and 5-Fluorouracil. (a) Preoperative enhanced CT revealed lesions located in the dome of the right hepatic lobe with uneven enhancement. (b-c) Intraoperative DSA angiography showed rich tumor vasculature, which significantly decreased after embolization with CSMs mixed with iodized oil. (d-f) Follow-up enhanced CT at 1, 3, and 6 months postoperatively revealed iodized oil deposition within the tumor, with gradual reduction in volume and absence of obvious enhancing components, suggesting complete remission.

**Fig. 3:**
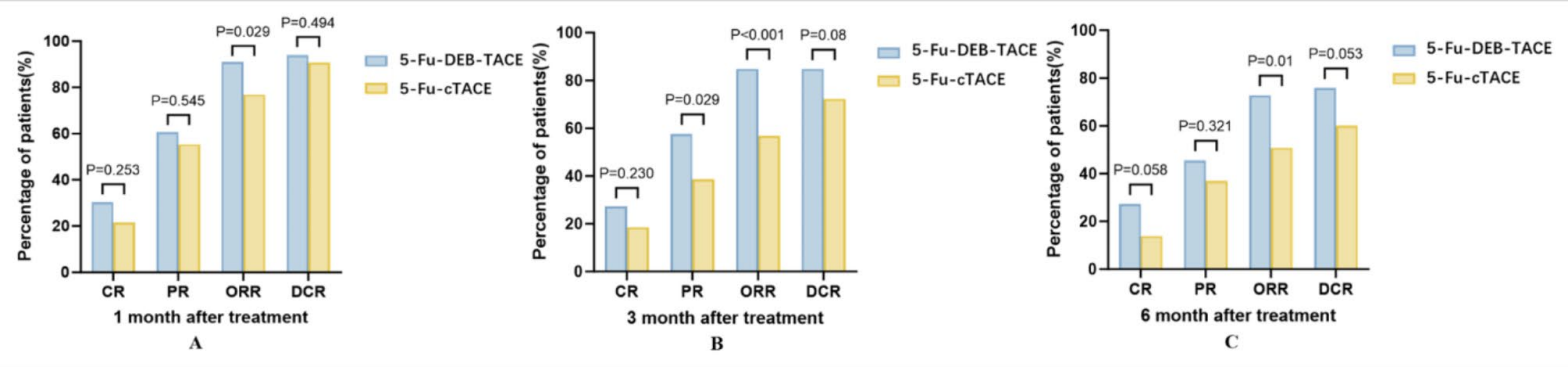
Tumor response at 1st, 3rd, and 6th month after treatment. ORR and DCR evaluating by modified Response Evaluation Criteria in Solid Tumors (mRECIST) in 5-Fu-DEB-TACE group and 5-Fu-cTACE group. The comparison of ORR and DCR at 1 month (A), 3 months (B), 6 months (C) after therapy between 5-Fu-DEB-TACE group and 5-Fu-cTACE group. CR = complete response, PR = partial response, SD = stable disease, PD = progressive disease. ORR: objective response rate, DCR: disease control rate.

The tumor response was assessed by experienced radiologists according to mRECIST at 1, 3, and 6 months after treatment (Table 3; Fig. 2). The ORR at 1 month after treatment in the 5-Fu-DEB-TACE group was 90.9% (60/66), while that in the 5-Fu-cTACE group was only 76.9% (50/65). The difference in ORR between the two groups was statistically significant (*P* = 0.029). However, the DCR (93.9% in the 5-Fu-DEB-TACE group vs. 90.8% in the 5-Fu-cTACE group, *P* = 0.494) was not significantly different (Fig. 3A). At 3 months after treatment, the 5-Fu-DEB-TACE group had a higher ORR (84.8% vs. 56.9%, *P* < 0.001) and DCR (84.8% vs. 72.3%, *P* = 0.08) (Fig. 3B). The ORR at 6 months was still higher in the 5-Fu-DEB-TACE group than in the 5-Fu-cTACE group (72.7% vs. 50.8%, *P* = 0.01), but the DCR was not significantly different (75.8% vs. 60.0%, *P* = 0.053) (Fig. 3C).

**Table 3:** Tumor response assessed at M1, M3 and M6 after treatment between 5-Fu-DEB-TACE group and 5-Fu-cTACE group.

	M1			M3			M6		
	5-Fu-DEB-TACE	5-Fu-cTACE	*p*-Value	5-Fu-DEB-TACE	5-Fu-cTACE	*p*-Value	5-Fu-DEB-TACE	5-Fu-cTACE	*p*-Value
CR	20(30.3%)	14(21.5%)	0.253	18(27.3%)	12(18.5%)	0.230	18(27.3%)	9(13.8%)	0.058
PR	40(60.6%)	36(55.4%)	0.545	38(57.6%)	25(38.5%)	0.029	30(45.5%)	24(36.9%)	0.321
SD	2(3%)	9(13.8%)	0.026	0(0)	10(15.4%)	<0.001	2(3%)	6(9.2%)	0.138
PD	4(6.1%)	6(9.2%)	0.494	10(15.1%)	18(27.6%)	0.052	16(24.2%)	26(40.1%)	0.053
ORR	60(90.9%)	50(76.9%)	0.029	56(84.8%)	37(56.9%)	<0.001	48(72.7%)	33(50.8%)	0.01
DCR	62(93.9%)	59(90.8%)	0.494	56(84.8%)	47(72.3%)	0.08	50(75.8%)	39(60%)	0.053

P value < 0.05 was considered significant. M1, 1 month; M3, 3 months; M6, 6 months; DEB-TACE, drug-eluting bead transarterial chemoembolization; cTACE, conventional transarterial chemo-embolization; 5-Fu, 5-Fluorouracil; CR, complete response; PR, partial response; SD, stable disease; ORR, objective response rate; DCR, disease control rate.

### Progression-free survival

**Fig. 4:**
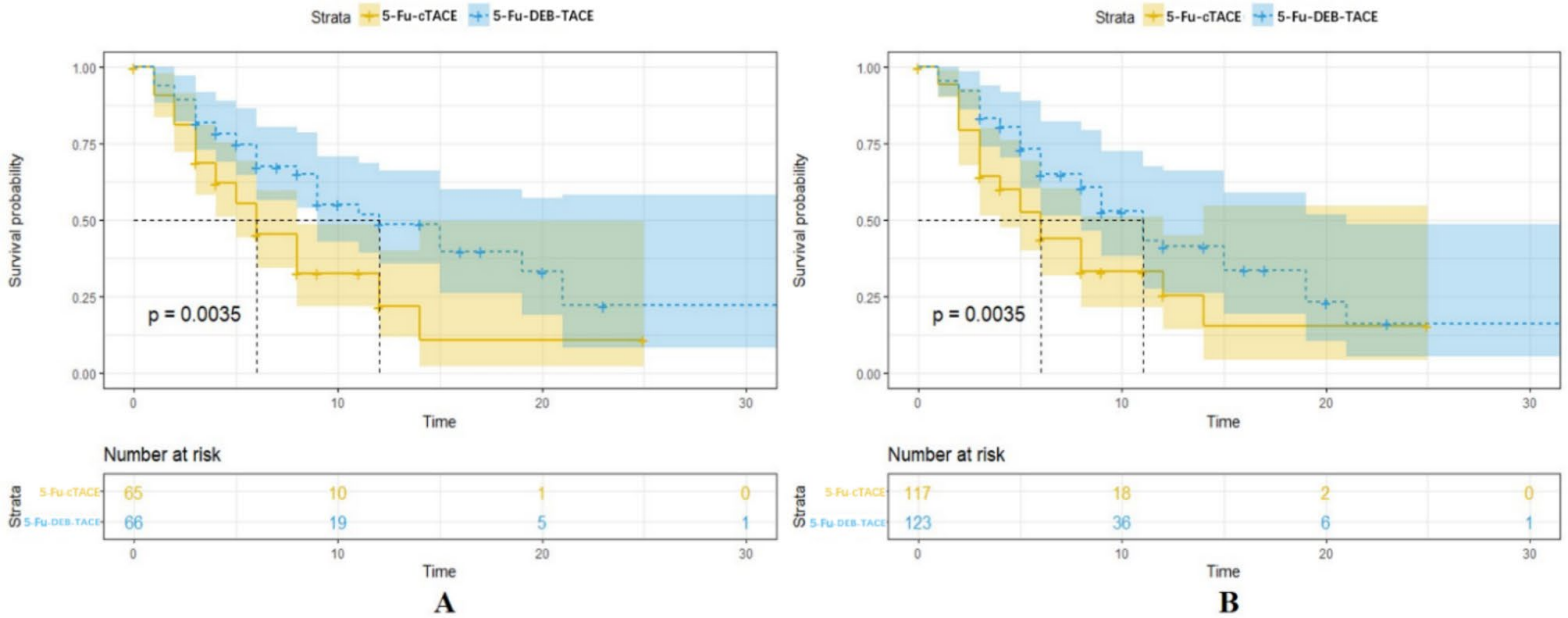
Kaplan-Meier curves demonstrating PFS before and after propensity score weighting. (A) Before propensity score weighting, the median PFS before propensity score weighting was 12 months (95%CI: 9, NA) in the 5-Fu-DEB-TACE group and 6 months (95%CI: 5,8) in the 5-Fu-cTACE group. (B) After propensity score weighting, the median PFS of 5-Fu-DEB-TACE group and 5-Fu-cTACE group were 11 months (95%CI: 8,21) and 6 months (95%CI: 4,12). DEB-TACE, drug-eluting beads TACE; cTACE, conventional transarterial chemoembolization.

At follow-up, the median PFS before propensity score weighting was 12 months (95% CI: 9, NA) in the 5-Fu-DEB-TACE group and 6 months (95% CI: 5, 8) in the 5-Fu-cTACE group. The survival curves of the two groups are shown in Fig. 4A, and the difference between the two groups was statistically significant (*P* = 0.004 according to the log rank test). After propensity score weighting, the median PFS of the 5-Fu-DEB-TACE group and 5-Fu-cTACE group were 11 months (95% CI: 8, 21) and 6 months (95% CI: 4, 12), respectively. The survival curve is shown in Fig. 4B, and the difference between the two groups was statistically significant (*P* = 0.004 according to the log rank test).

### Factors affecting PFS

Cox proportional hazards regression analysis was performed on all 131 HCC patients treated with TACE combined with 5-Fu, and factors significantly associated with PFS in the univariate analysis were included in the multivariate analysis (Table 4). After propensity score weighting, the univariate regression analysis showed that 5-Fu-DEB-TACE was associated with a longer PFS (HR = 0.570, *P* = 0.041), while Child‒Pugh stage B (HR = 3.304, *P* < 0.001), MELD score (HR = 4.656, *P* < 0.001), BCLC stage B (HR = 2.639, *P* = 0.016), BCLC stage C (HR = 3.166, *P* = 0.003), vascular invasion (HR = 1.789, *P* = 0.034) and ascitic fluid (HR = 2.597, *P* < 0.001) were associated with a shorter PFS. Multivariate regression analysis revealed that 5-Fu-DEB-TACE (HR = 0.590, *P* = 0.044), MELD intermediate risk (HR = 2.470, *P* = 0.010), BCLC stage B (HR = 2.303, *P* = 0.036), BCLC stage C (HR = 3.354, *P* = 0.001) and ascitic fluid (HR = 2.004, *P* = 0.046) were independent predictors of PFS.

**Table 4:** Cox proportional hazard regression analysis for progression-free survival after propensity score weighting.

Parameter	Univariate Analysis Hazard Ratio (95% CI)	*p*-Value	Multivariate Analysis Hazard Ratio (95% CI)	*p*-Value
TACE method 5-Fu-cTACE 5-Fu-DEB-TACE	1.00 0.57(0.33–0.97)	0.041	1.00 0.59(0.35–0.98)	0.044
Gender		0.793		
Female	1.00			
Male	0.92(0.52–1.62)			
Age(years)	1.01(0.99–1.03)	0.227		
Diabetes No Yes	1.00 1.39(0.75–2.58)	0.284		
History of HB No Yes	1.00 1.07(0.64–1.81)	0.774		
Liver Cirrhosis No Yes	1.00 0.91(0.58–2.02)	0.790		
AFP(µg/L) <400 ≥ 400	1.00 0.97(0.52–1.80)	0.937		
PIVKA-II(mAU/ml) <40 ≥ 40	1.00 1.61(0.85-3.05)	0.141		
Ascites No Yes	1.00 2.59(1.51–4.46)	<0.001	1.00 2.00(1.01–3.96)	0.046
MELD Low risk Medium risk	1.00 4.65(3.01–7.19)	<0.001	1.00 2.47(1.23–4.93)	0.010
ALBI Level 1 Level 2	1.00 1.39(0.80–2.41)	0.239		
Child-pugh Stage A B	1.00 3.30(1.88–5.80)	<0.001	1.00 1.82(0.77–4.32)	0.17
ECOG performance status 0 1 2	1.00 1.38(0.71–2.67) 1.04(0.50–2.18)	0.339 0.905		
BCLC Stage A B C	1.00 2.63(1.19–5.84) 3.16(1.45–6.87)	0.016 0.003	1.00 2.30(1.05–5.02) 3.35(1.56–7.19)	0.036 0.001
Largest nodule size (cm) ≥ 5 <5	1.00 1.33(0.72–2.66)	0.325		
Tumor multiplicity Unifocal Multifocal	1.00 1.25(0.74–2.12)	0.388		
Vascular Invasion No Yes	1.00 1.78(1.04–3.07)	0.034	1.00 1.48(0.88–2.47)	0.13
Lymphatic Metastasis No Yes	1.00 1.42(0.84–2.41)	0.182		
Extrahepatic metastasis No Yes	1.00 1.63(0.93–2.84)	0.082		

DEB-TACE, drug-eluting beads trans-arterial chemoembolization; cTACE, conventional trans-arterial chemoembolization; 5-Fu, 5-Fluorouracil; HB, hepatitis B; AFP, alpha-fetoprotein; PIVKA-II, Protein Induced by Vitamin K Absence or Antagonist-II; MELD, Model for End-Stage Liver Disease; ALBI, Albumin-Bilirubin; ECOG, Eastern Oncology Group; BCLC, Barcelona Clinic Liver cancer.

### Subgroup analysis

**Fig. 5:**
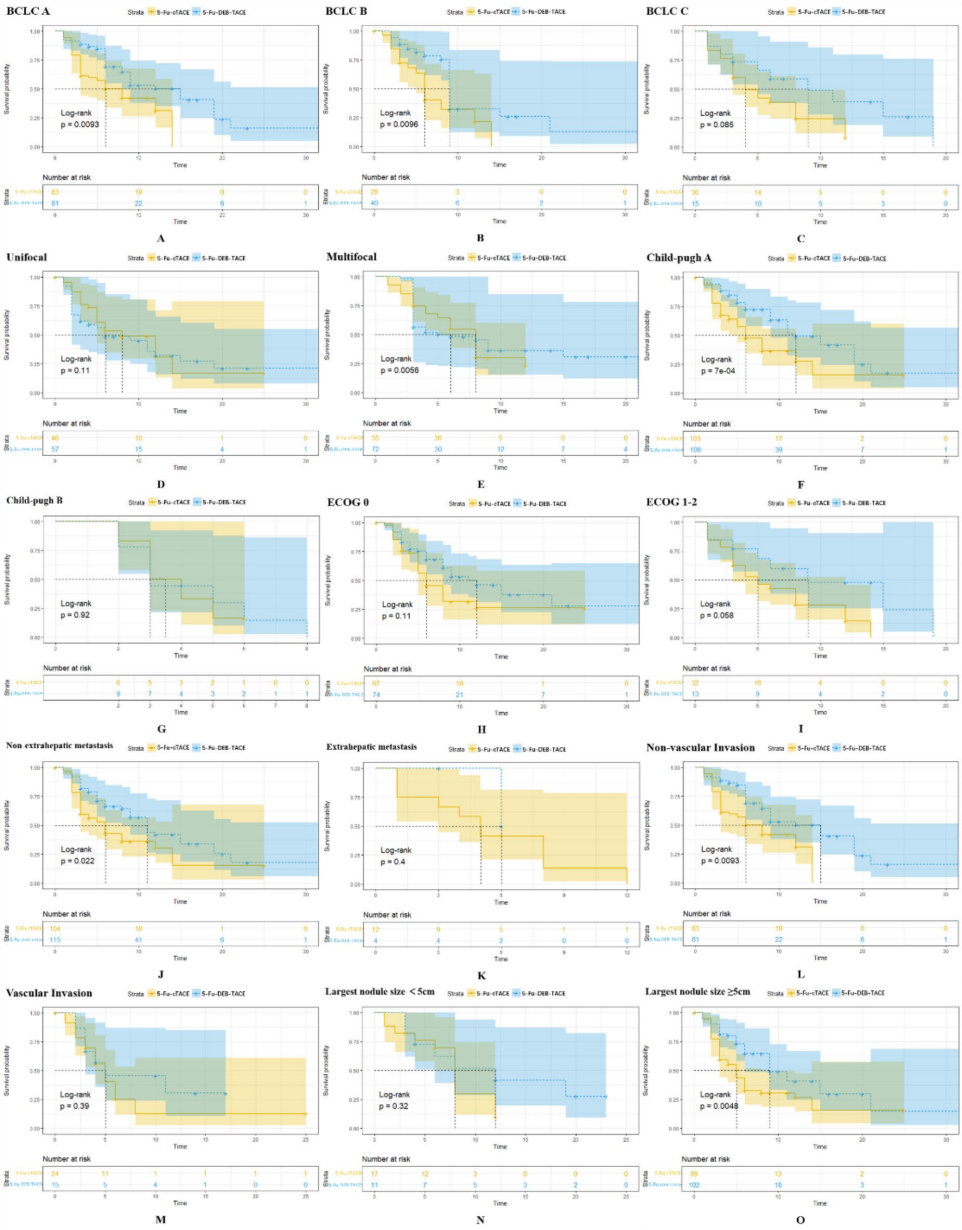
Subgroup analyses of PFS. The 5-Fu-DEB-TACE group showed a significantly longer PFS in the subgroup of patients with BCLC stage A (A), BCLC stage (B), multifocal (D), Child-Pugh stage A (F), non- extrahepatic metastasis (J), non-vascular invasion (L), and with a largest tumor diameter ≥ 5 cm (O), whereas no significant difference was found in the other subgroups (C, E, G-I, K, M, N). Kaplan–Meier method and log-rank test were implemented to evaluate the difference of survival between two groups. *P* < 0.05 was considered significant. DEB-TACE, drug-eluting beads TACE; cTACE, conventional transarterial chemoembolization. BCLC, Barcelona Clinic Liver cancer.

Perform subgroup survival analysis based on BCLC staging, Child-Pugh classification, ECOG score, number of tumors, largest tumor diameter, presence of extrahepatic metastasis, and vascular invasion. The PFS was compared between the 5-Fu-DEB-TACE and 5-Fu-cTACE groups in various subgroups by the Kaplan-Meier method and log-rank test, the results show that patients receiving 5-Fu-DEB-TACE have better PFS compared to those receiving 5-Fu-cTACE in the BCLC stage A group (*P* = 0.0093), BCLC stage B group (*P* = 0.0096), multifocal group (*P* = 0.0056), Child-Pugh stage A group (*P*<0.001), non- extrahepatic metastasis group (*P* = 0.022), non-vascular invasion group (*P* = 0.0093), and the group with a largest tumor diameter ≥ 5 cm (*P* = 0.0048) (Fig. 5).

The chi-square test was used to compare the ORR and DCR across subgroups. The results indicate that at M1, the 5-Fu-DEB-TACE group presented with elevated ORR in patients with Child-pugh stage A (*P* = 0.038). At M3, the 5-Fu-DEB-TACE group presented with elevated ORR in patients with BCLC stage B (*P* = 0.004), Child-pugh stage A (*P*<0.001), ECOG 0 (*P* = 0.002), single lesion (*P* = 0.003), multifocal lesions (P = 0.037), no extrahepatic metastasis (*P* = 0.001), no vascular invasion (*P* = 0.002), largest size ≥ 5 cm (*P*<0.001). At M6, the ORR was increased in the 5-Fu-DEB-TACE group than that in the 5-Fu-cTACE group in patients with Child-pugh stage A (*P* = 0.005), single lesion (*P* = 0.007), no extrahepatic metastasis (*P* = 0.028), no vascular Invasion (*P* = 0.012), largest size ≥ 5 cm (*P* = 0.003). Regarding DCR, there is no significant difference between the 5-Fu-DEB-TACE and 5-Fu-cTACE groups at M1. At M3, the 5-Fu-DEB-TACE group shows higher DCR in patients with BCLC B stage (*P* = 0.022), Child-Pugh A grade (*P* =0.023), and largest tumor diameter ≥ 5 cm (*P* = 0.042). At M6, patients in the 5-Fu-DEB-TACE group with Child-Pugh A grade (*P* = 0.028), unifocal lesion (*P* = 0.033), and largest diameter ≥ 5 cm (*P* = 0.010) have higher DCR (Table 5).

**Table 5:** Comparison of ORR and DCR in subgroup analysis.

	M1			M3			M6		
	5-Fu-DEB-TACE	5-Fu-cTACE	*p*-Value	5-Fu-DEB-TACE	5-Fu-cTACE	*p*-Value	5-Fu-DEB-TACE	5-Fu-cTACE	*p*-Value
**BCLC A**									
ORR	21(91.3%)	11(100%)	0.313	20(87%)	8(72.7%)	0.309	16(69.6%)	9(81.8%)	0.449
DCR	21(91.3%)	11(100%)	0.313	20(87%)	10(90.9%)	0.738	17(73.9%)	10(90.9%)	0.252
**BCLC B**									
ORR	26(92.9%)	19(79.2%)	0.149	25(89.3%)	13(54.2%)	**0.004**	22(78.6%)	13(54.2%)	0.061
DCR	28(100%)	23(95.8%)	0.275	25(89.3%)	15(62.5%)	**0.022**	22(78.6%)	14(58.3%)	0.115
**BCLC C**									
ORR	13(86.7%)	20(66.7%)	0.153	11(73.3%)	16(53.3%)	0.197	10(66.7%)	11(36.7%)	0.057
DCR	13(86.7%)	25(83.3%)	0.771	11(73.3%)	22(73.3%)	1.000	11(73.3%)	15(50%)	0.135
**Child-pugh A**									
ORR	53(93%)	47(79.7%)	**0.038**	51(89.5%)	36(61%)	**<0.001**	45(78.9%)	32(54.2%)	**0.005**
DCR	53(93%)	53(89.8%)	0.545	51(89.5%)	43(72.9%)	**0.023**	47(82.5%)	38(64.4%)	**0.028**
**Child-pugh B**									
ORR	7(77.8%)	3(50%)	0.264	5(55.6%)	1(16.7%)	0.132	3(33.3%)	1(16.7%)	0.475
DCR	9(100%)	6(100%)	-	5(55.6%)	4(66.7%)	0.667	3(33.3%)	1(16.7%)	0.475
**ECOG 0**									
ORR	49(92.5%)	28(84.8%)	0.263	46(86.8%)	19(57.6%)	**0.002**	40(75.5%)	21(63.3%)	0.240
DCR	51(96.2%)	32(97%)	0.855	46(86.8%)	23(69.7%)	0.053	41(77.4%)	22(66.7%)	0.276
**ECOG 1–2**									
ORR	11(84.6%)	22(68.8%)	0.275	10(76.9%)	18(56.3%)	0.195	8(61.5%)	12(37.5%)	0.141
DCR	11(84.6%)	27(84.4%)	0.984	10(76.9%)	24(75%)	0.892	9(69.2%)	17(53.1%)	0.321
**Unifocal**									
ORR	31(93.9%)	29(78.4%)	0.063	30(90.9%)	22(59.5%)	**0.003**	27(81.8%)	19(51.4%)	**0.007**
DCR	33(100%)	33(89.3%)	0.052	30(90.9%)	28(75.7%)	0.091	28(84.8%)	23(62.2%)	**0.033**
**Multifocal**									
ORR	29(87.9%)	21(75%)	0.192	26(78.8%)	15(53.6%)	**0.037**	21(63.6%)	14(50%)	0.283
DCR	29(87.9%)	26(92.9%)	0.515	26(78.8%)	19(67.9%)	0.333	22(66.7%)	16(57.1%)	0.444
**Non-extrahepatic metastasis**									
ORR	56(90.3%)	42(79.2%)	0.095	53(85.5%)	31(58.5%)	**0.001**	45(72.6%)	28(52.8%)	**0.028**
DCR	58(93.5%)	50(94.3%)	0.172	53(85.5%)	39(73.6%)	0.112	47(75.8%)	34(64.2%)	0.172
**Extrahepatic metastasis**									
ORR	4(100%)	8(66.7%)	0.182	3(75%)	6(50%)	0.383	3(75%)	5(41.7%)	0.248
DCR	4(100%)	9(75%)	0.267	3(75%)	8(66.7%)	0.755	3(75%)	5(41.7%)	0.248
**Non-vascular Invasion**									
ORR	46(90.2%)	32(78%)	0.107	47(92.2%)	27(65.9%)	**0.002**	40(78.4%)	22(53.7%)	**0.012**
DCR	47(92.2%)	37(90.2%)	0.746	47(92.2%)	32(78%)	0.053	42(82.4%)	27(65.9%)	0.069
**Vascular Invasion**									
ORR	14(93.3%)	18(75%)	0.147	9(60%)	10(41.7%)	0.265	8(53.3%)	11(45.8%)	0.648
DCR	15(100%)	22(91.7%)	0.251	9(60%)	15(62.5%)	0.876	8(53.3%)	12(50%)	0.839
**Largest size <5 cm**									
ORR	11(100%)	14(82.4%)	0.140	9(81.8%)	12(70.6%)	0.503	7(63.6%)	11(64.7%)	0.954
DCR	11(100%)	15(88.2%)	0.238	9(81.8%)	14(82.4%)	0.971	8(72.7%)	14(82.4%)	0.544
**Largest size ≥ 5 cm**									
ORR	49(89.1%)	36(75%)	0.060	47(85.5%)	25(52.1%)	**<0.001**	41(74.5%)	22(45.8%)	**0.003**
DCR	51(92.7%)	44(91.7%)	0.841	47(85.5%)	33(68.8%)	**0.042**	42(76.4%)	25(52.1%)	**0.010**

P value < 0.05 was considered significant, and the significant results were shown in boldface. M1, 1 month; M3, 3 months; M6, 6 months; DEB-TACE, drug-eluting bead transarterial chemoembolization; cTACE, conventional transarterial chemo-embolization; 5-Fu, 5-Fluorouracil; ECOG, Eastern Oncology Group; BCLC, Barcelona Clinic Liver cancer; ORR, objective response rate; DCR, disease control rate.

### Adverse events

Adverse events and biochemical toxicity observed after 5-Fu-DEB-TACE and 5-Fu-cTACE are shown in Table 6. The vast majority of adverse events were grade I-II, with only one grade II-IV event occurring in the 5-Fu-DEB-TACE group (following surgery, a patient developed a liver abscess and exhibited an improvement after receiving aggressive anti-infective therapy). The most common adverse events were postembolization syndromes, such as nausea, vomiting, fever, and abdominal pain. With the exception of abdominal pain (5-Fu-DEB-TACE group, 48/66 (72.7%) vs. 5-Fu-cTACE group, 31/65 (47.7%), *P* = 0.003), no significant differences were observed in the remaining symptoms between the two groups. In terms of biochemical toxicity, the patients in the 5-Fu-DEB-TACE group had significantly less liver function damage than those in the 5-Fu-cTACE group (44/66 (66.6%) vs. 54/65 (83.1%), *P* = 0.031).

**Table 6:** Adverse reactions after 5-Fu-DEB-TACE and 5-Fu-cTACE treatments.

Adverse event	5-Fu-DEB-TACE				5-Fu-cTACE				*p*-Value
	I	II	III	IV	I	II	III	IV	
Nausea	13(19.6%)	1(1.5%)	0	0	14(21.5%)	0	0	0	0.964
Vomiting	10(15.1%)	2(3.0%)	0	0	11(16.9%)	0	0	0	0.850
Fever	7(10.6%)	6(9.1%)	0	0	10(15.3%)	1(1.5%)	0	0	0.682
Abdominal pain	38(57.5%)	10(15.1%)	0	0	23(35.3%)	8(12.3%)	0	0	0.003
Hepatotoxicity	25(37.8%)	19(28.7%)	0	0	32(49.2%)	22(33.8%)	0	0	0.031
Liver abscess	0	0	1	0	0	0	0	0	-

DEB-TACE, drug-eluting beads trans-arterial chemoembolization; cTACE, conventional trans-arterial chemoembolization; 5-Fu, 5-Fluorouracil.

## Discussion

In this study, we compared the efficacy and safety of 5-Fu-DEB-TACE and 5-Fu-cTACE for the treatment of unresectable HCC, and the main findings were as follows: (1) The patients in the 5-Fu-DEB-TACE group had a better tumor response than those in the 5-Fu-cTACE group. (2) Patients in the 5-Fu-DEB-TACE group had a longer PFS than those in the 5-Fu-cTACE group. (3) The BCLC stage, MELD score, and ascites were found to be independent predictive factors for PFS. (4) 5-Fu-DEB-TACE had less of an effect on liver function damage but was associated with a higher incidence of pain during hospitalization.

Although previous studies have indicated the superiority of CSMs^18–21^, only a few retrospective studies have compared the efficacy and safety of DEB-TACE using CSMs and cTACE^29–31^. Because retrospective observational studies are limited by various biases and confounding factors, reliable comparisons of results between experimental and control groups are limited. Propensity score weighting can balance confounding factors, eliminate confounding factors, improve comparability between groups, and directly estimate the treatment effect^32^. Therefore, after balancing confounding factors, we compared the short-term efficacy of these two TACE treatments combined with 5-Fu in patients with HCC and found that 5-Fu-DEB-TACE using CSMs was more effective than 5-Fu-cTACE at 1, 3, and 6 months posttreatment. The ORR and DCR of the 5-Fu-DEB-TACE group at 1, 3, and 6 months were 90.9%, 84.8%, and 72.7% and 93.9%, 84.8%, and 75.8%, respectively. In the 5-Fu-cTACE group, the ORR and DCR were 76.9%, 56.9%, and 50.8% and 90.8%, 72.3%, and 60%, respectively. This finding is consistent with existing research results. (Liang et al. reviewed 335 patients with HCC who underwent treatment with DEB-TACE using CSMs or cTACE. The results indicated that the ORR and DCR at 1, 3, and 6 months posttreatment were 71.9%, 76.8%, and 70.4% and 89.7%, 82.9%, and 88.9%, respectively, for the DEB-TACE group using CSMs, while the ORR and DCR of the cTACE group were 46.8%, 45.5%, and 46.7% and 87.1%, 83.6%, and 80.0%, respectively.) This result may be because CSMs can continuously release drugs inside the tumor, preventing a reduction in the local drug concentration; moreover, CSMs with a diameter of 100–300 μm can be embolized to the distal end of the tumor blood vessels, leading to a more thorough blockade of the tumor blood supply. Our study revealed that the efficacy of both treatment methods was superior to that of previous methods. The reasons for these results may be the increasing detection rate of HCC in recent years, resulting in fewer patients in the terminal stage being included in this study. Another reason may be that we infused 5-fluorouracil into the hepatic artery of patients before embolization. A recent study involving the combination of HAIC therapy with fluorouracil and oxaliplatin in 157 of 315 postoperative HCC patients revealed that the median disease-free survival (DFS) was 20.3 months in the treatment group versus 10.0 months in the control group. The overall survival rates at 1 year, 2 years, and 3 years were 93.8%, 86.4%, and 80.4%, respectively, for the treatment group and 92.0%, 86.0%, and 74.9%, respectively, for the control group. This research revealed that fluorouracil has good effectiveness in treating HCC with combined microvascular invasion^33^. Research by Kim et al. and Liu et al. on post-embolization chemotherapy showed that survival was extended compared to cTACE, with median OS of 9.7 months and 8 months, respectively, without increasing the risk of post-treatment complications^34,35^. However, since some tumor blood vessels are blocked, the distribution and penetration of the drug may be affected, potentially reducing drug concentration and therapeutic efficacy in the tumor area. On the other hand, administering drugs before embolization not only increases drug concentration within the lesion and extends the duration of drug action but also helps reduce systemic distribution to lower side effects. Li et al. administered 100–150 mg of oxaliplatin and 1000 mg of 5-Fu before TACE in patients with HCC having a tumor diameter ≥ 10 cm. Even with high tumor burden, the final OS improved (median OS of 10.3 months) with good safety^36^. However, there is limited research on the combination of 5-fluorouracil and TACE, and there is no unified consensus on the timing of intra-arterial chemotherapy drugs. More studies are needed in the future to confirm its efficacy and safety.

Regarding progression-free survival, some studies have suggested that DEB-TACE is associated with a longer PFS, while other studies have indicated that it is similar to cTACE in terms of controlling tumor progression. Therefore, controversy still exists regarding whether DEB-TACE is more effective than cTACE in improving PFS for HCC patients^14–17^, and the same applies to DEB-TACE combined with CSMs^20,30^. We conducted the current study to answer this question, and the results showed that compared to the 5-Fu-cTACE group, the 5-Fu-DEB-TACE group had a longer median PFS. Furthermore, the multivariate Cox proportional hazards regression model showed that 5-Fu-DEB-TACE was an independent predictor of a longer PFS, further supporting our findings that 5-Fu-DEB-TACE combined with CSMs is better at controlling tumor progression than 5-Fu-cTACE. The possible reason for this result is that the CSM used in DEB-TACE can deliver chemotherapeutic drugs directly to the tumor site, ensuring more uniform and sustained drug release^37^. This property allows a longer duration of drug action to improve local tumor control and reduce the risk of recurrence. Moreover, CSMs with particle sizes ranging from 100 to 300 μm can more easily embolize to the periphery of tumor blood vessels, leading to more extensive tumor necrosis and preventing recurrence. In addition to 5-Fu-DEB-TACE, our study revealed that the MELD score and BCLC stage were independent predictors of PFS. The BCLC stage is a clinically recognized prognostic model for HCC, and the MELD score represents the extent of liver damage. Previous research has documented its reliable predictive value for all advanced liver diseases^38^, and the results of our study also support this conclusion. The MELD score takes into account several indicators of liver function, including total bilirubin, the international normalized ratio (INR), and creatinine levels, thus reflecting liver function and overall metabolic conditions. The more aggressive the tumor is, the more severe the deterioration in liver function. Therefore, a high MELD score often indicates a more severe tumor and signifies increased surgical risk, necessitating more careful preoperative preparation and postoperative monitoring. Additionally, a multifactor Cox proportional hazards regression analysis indicated that ascites is an independent predictor of a shorter PFS. Previous studies have explored the predictive value of ascites for the prognosis of HCC^39^, and our results align with these findings. This finding could be because ascites is also an indication of poor liver function. Even if liver function tests are within the normal range, the presence of ascites suggests a marginal liver reserve, making the liver more sensitive to external stressors or further damage. Early research also suggested that HCC patients with ascites have a greater risk of ischaemic injury after surgery; hence, TACE treatment is not recommended^40^. Unlike in previous studies, we observed that the tumor burden did not have a significant impact on prognosis^41,42^, which may be attributed to the small sample size and inadequate follow-up time.

In unresectable HCC, tumor burden and liver function have a significant impact on prognosis. Patients with a tumor burden that beyond up-to-seven criteria or with poor liver function reserves are considered TACE-unsuitable^43,44^. Therefore, personalized treatment plans are crucial. We conducted a subgroup analysis based on liver function and tumor burden to assess the outcomes of patients with various characteristics after TACE treatment. The results show that, apart from the maximum tumor diameter, 5-Fu-DEB-TACE treatment demonstrates superior PFS, ORR, and DCR compared to 5-Fu-cTACE in cases with lower tumor burden and better liver function. In contrast, when tumor burden is higher and liver function is poorer, the efficacy of TACE is reduced, which is consistent with previous research findings^44,45^. The possible reasons are that when the tumor burden is high, TACE treatment may not be able to comprehensively cover all tumor lesions, and the increased blood flow can affect the concentration and effectiveness of the drugs. Additionally, under the hypoxic and chemotherapeutic effects induced by TACE, surviving tumor cells in high tumor burden lesions often transform into a more invasive phenotype and develop resistance to TACE^46,47^. Regarding tumor diameter, the study by Kim et al. suggests that a tumor diameter >5 cm is a significant predictor of shorter OS with cTACE, indicating that DEB-TACE may be more suitable for larger tumors^48^. Our research found that in patients with a tumor diameter ≥ 5 cm, the 5-Fu-DEB-TACE group had better PFS, ORR, DCR compared to the 5-Fu-cTACE group, supporting this conclusion. This may be because the CSM used in DEB-TACE provides more targeted, uniform, and sustained drug release along with stronger embolization, which meets the higher drug concentration and ischemic effect requirements for large tumors^6,37^. However, Lee et al. found no significant difference between the two TACE methods in patients with tumors greater than 5 cm in diameter^49^. Vesselle et al. observed that the efficacy of both TACE methods significantly decreased with tumors larger than 5 cm^50^. Whether DEB-TACE has a clear advantage in treating large lesions still requires further validation through prospective studies.

Regarding safety comparisons, no treatment-related deaths occurred in this study. Postoperative complications in both groups mainly manifested as embolism syndrome, primarily at grades I-II. Apart from a higher incidence of abdominal pain in the 5-Fu-DEB-TACE group, no significant differences were observed in other symptoms. This result may be because the embolic capability of CSMs is stronger than that of the iodized oil used in conventional TACE, leading to a more pronounced tumor ischaemic effect in the short term. Additionally, the results indicate that most patients in both groups experienced elevated transaminase levels after treatment, with the 5-Fu-DEB-TACE group having a relatively lower transaminase level, possibly due to the different drug release mechanisms of CSMs. The main reasons for postoperative transaminase elevations include liver tissue damage and impaired liver function. CSMs have better vascular targeting ability, causing less damage to the surrounding normal liver tissue during embolization and releasing fewer transaminases. In addition, DEB-TACE using CSMs can continuously release drugs within the tumor, reducing the burden on the liver to process metabolic byproducts and drug residues after treatment, resulting in a lower impact on transaminase metabolism. The transaminase levels in these patients mostly decreased approximately one month after treatment. Therefore, we believe that the safety of 5-Fu-DEB-TACE using CSMs is comparable to that of 5-Fu-cTACE and that neither approach results in long-term liver function abnormalities.

The selection of the microsphere size is crucial for TACE treatment. In this study, microspheres with a size range of 100–300 μm were chosen for the following reasons. First, theoretically, smaller microspheres are distributed more evenly within the tumor and deposit more in the peripheral blood vessels, leading to a larger area of tumor necrosis. Previous studies have indicated that compared to microspheres ranging from 100 to 300 μm, those ranging from 70 to 150 μm exhibit a better tumor response to DEB-TACE treatment while maintaining similar safety profiles^51^. However, excessively small microspheres may result in incomplete or ectopic embolization and can impact drug loading and release. A study comparing the drug loading and release efficiency of CSM ranging from 50 to 150 μm, 100 to 300 μm, and 300 to 500 μm showed that 50–150 μm microspheres have a higher drug loading rate, while 300–500 μm microspheres exhibit advantages in sustained drug release^52^. After considering these factors, we chose to use CSMs with a size range of 100–300 μm.

This study has several limitations. First, the follow-up period was insufficient, and some patients had not yet experienced significant events despite showing survival benefits. Further observation is required in the future. Second, although the tumor response was evaluated by multiple experienced radiologists, factors such as iodized oil deposition and irregular necrosis make it challenging to make accurate assessments based on mRECIST. Last, this study was conducted at a single centre with a limited sample size, indicating the necessity of expanding the cohort for future studies.

## Conclusions

In conclusion, this propensity score-weighted study indicates that in patients with unresectable HCC, 5-Fu-DEB-TACE using CSMs offers better efficacy, a longer PFS, and reduced liver function damage than 5-Fu-cTACE, especially in cases with a maximum tumor diameter ≥ 5 cm, good liver function reserve, and a relatively lower tumor burden, albeit with a slightly increased probability of abdominal pain. The BCLC stage, MELD score, and presence of ascites were found to be independent predictive factors for PFS. Larger studies are needed to further validate its long-term effectiveness and clinical benefits.

## Data Availability

The data that support the findings of this study are available from the corresponding author upon reasonable request.
